# Adjuvant treatment with tumor-targeting *Salmonella typhimurium* A1-R reduces recurrence and increases survival after liver metastasis resection in an orthotopic nude mouse model

**DOI:** 10.18632/oncotarget.6170

**Published:** 2015-10-19

**Authors:** Takashi Murakami, Yukihiko Hiroshima, Ming Zhao, Yong Zhang, Takashi Chishima, Kuniya Tanaka, Michael Bouvet, Itaru Endo, Robert M. Hoffman

**Affiliations:** ^1^ AntiCancer, Inc., San Diego, California, USA; ^2^ Department of Surgery, University of California, San Diego, California, USA; ^3^ Department of Gastroenterological Surgery, Graduate School of Medicine, Yokohama City University, Japan

**Keywords:** liver metastasis, colon cancer, RFP, nude mice, orthotopic models

## Abstract

Colon cancer liver metastasis is often the lethal aspect of this disease. Well-isolated metastases are candidates for surgical resection, but recurrence is common. Better adjuvant treatment is therefore needed to reduce or prevent recurrence. In the present study, HT-29 human colon cancer cells expressing red fluorescent protein (RFP) were used to establish liver metastases in nude mice. Mice with a single liver metastasis were randomized into bright-light surgery (BLS) or the combination of BLS and adjuvant treatment with tumor-targeting *S. typhimurium* A1-R. Residual tumor fluorescence after BLS was clearly visualized at high magnification by fluorescence imaging. Adjuvant treatment with *S. typhimurium* A1-R was highly effective to increase survival and disease-free survival after BLS of liver metastasis. The results suggest the future clinical potential of adjuvant *S. typhimurium* A1-R treatment after liver metastasis resection.

## INTRODUCTION

Colon cancer liver metastasis is often the lethal aspect of this disease [1]. Well-isolated metastases are candidates for surgical resection, but recurrence is common [2]. Better adjuvant treatment is therefore needed to reduce or prevent recurrence.

Bacterial therapy of cancer has a long history [3-5]. The bacteria, now known as *Streptococcus pyogenes* [3-5], was found in cancer patients who had remission and was used for therapy in the late 19^th^ and early 20^th^ centuries [3-5], especially under William B. Coley.

Hoption Cann et al. [5] compared patient outcome with early bacterial treatment to modern chemotherapy and found the 10-year survival rates were comparable [5]. Beginning in the middle of the last century, bacteria were used for cancer therapy in animal models [6-18]. *Bifidobacterium* [18] and *Clostridium* [19], obligate anaerobes which replicate only in necrotic areas of tumors, had anti-tumor efficacy in mouse tumor models. Spores of *Clostridium novyi*, without its lethal toxin (*C. novyi* no toxin [NT]), were recently used in a patient with leiomyosarcoma causing a metastatic lesion to regress after intratumor administration [20].

*Salmonella typhimurium* (*S. typhimurium*) is a facultative anaerobe, which in contrast to obligate anaerobes, allows growth in the viable regions as well as necrotic regions of tumors [20]. *S. typhimurium-*VNP20009, with msbB and purI mutations, was found safe in a Phase I clinical trial of metastatic melanoma and renal carcinoma [21].

The tumor-targeting *S. typhimurium* A1-R strain developed by our laboratory has higher tumor colonization efficacy and antitumor efficacy than *S. typhimurium*-VNP20009 [22], possibly because it has fewer attenuation mutations. *S. typhimurium* A1-R is auxotrophic for Leu--Arg, which prevents it from mounting a continuous infection in normal tissues. *S. typhimurium* A1-R was able to inhibit or eradicate primary and metastatic tumors as monotherapy in nude mouse models of prostate [23, 24], breast [25-27], lung [28, 29], pancreatic [30-34], ovarian [35, 36] stomach [37], and cervical cancer [38], as well as sarcoma [39-41] and glioma [42,43], all of which are highly aggressive tumor models.

The present report demonstrates adjuvant treatment efficacy of *S. typhimurium* A1-R after bright-light surgery (BLS) of liver metastasis.

## RESULTS AND DISCUSSION

### BLS cannot resect the entire liver metastasis

**Figure 1:**
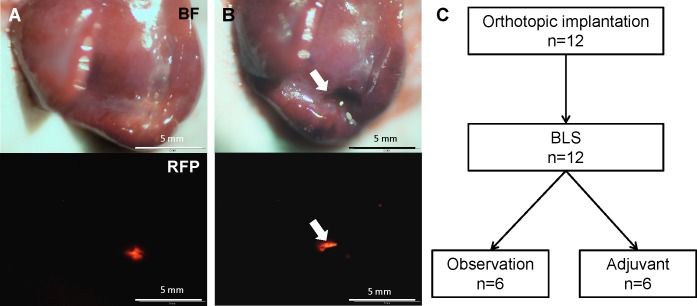
Efficacy of BLS alone on liver metastasis **A.** Tumor fluorescence was clearly detected before BLS. **B.** Tumor fluorescence still remained on the surgical resection bed (arrows) after BLS. **C.** Schematic diagram of the experimental design for adjuvant *S. typhimurium* A1-R treatment. Twelve mice were randomized into observation (n=6) and adjuvant groups (n=6). BF=bright field.

Residual tumor fluorescence was detected on the surgical resection bed after BLS of HT-29-RFP liver metastasis (Figure 1). There was no significant difference in residual tumor area between the control group (0.237 ± 0.094 mm^2^) and BLS group (0.250 ± 0.120 mm^2^).

### Adjuvant treatment with *S. typhimurium* A1-R increases survival after BLS

**Figure 2:**
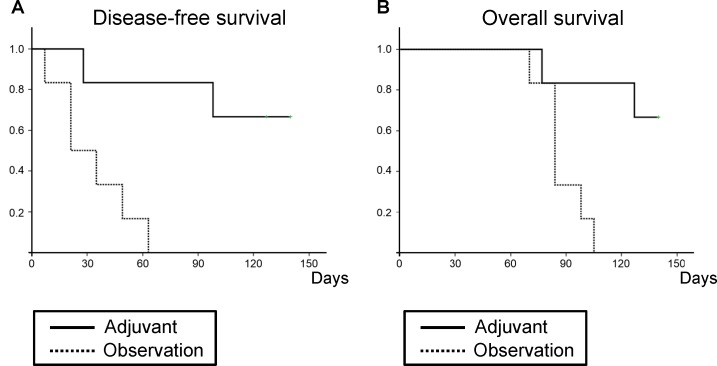
Efficacy of adjuvant *S. typhimurium* A1-R treatment on disease-free and overall survival according to the Kaplan-Meier method Mice in the adjuvant group had reduced recurrence (A) and increased survival (B) compared to those in the untreated control group.

Adjuvant *S. typhimurium* A1-R treatment after BLS significantly prolonged both disease-free (*P* = 0.005; Figure 2A) and overall survival (*P* = 0.010; Figure 2B).

Liver metastasis of colon cancer is the major lethal event of this disease. In many cases, diffuse liver metastasis is inoperable. However, isolated liver metastasis provides an opportunity for resection, but BLS very often results in residual cancer cells. The present report demonstrates that *S. typhimurium* A1-R can eradicate sufficient residual cancer cells after BLS to significantly increase disease-free survival and overall survival. Future experiments will also use *S. typhimurium* A1-R as neoadjuvant chemotherapy to convert inoperable tumors to those that are resectable.

In a recent study, we found that *Salmonella typhimurium* A1-R was active as monotherapy on liver metastasis in the orthotopic HT-29 mouse model. The results of that study demonstrated the potential of *S. typhimurium* A1-R targeting of liver metastasis [44].

The recurrence rate after liver metastasis resection is high, up to 75% [1, 2, 45]. Adjuvant therapy for colon cancer liver metastasis is usually based on 5-fluorouracil and oxaliplatin and it is not highly effective [1, 2, 45]. Therefore, novel approaches are necessary to improve adjuvant therapy of colon cancer liver metastasis.

The present study indicated that *S. typhimuirum* A1-R can be curative as adjuvant treatment for liver metastasis. Clinical trials of *S. typhimurium* A1-R for adjuvant therapy of patients with liver metastasis resection would have high potential.

Previously developed concepts and strategies of highly selective tumor targeting [46-51] can take advantage of bacterial targeting of tumors, including tissue-selective therapy which focuses on unique properties of normal and tumor tissues [46, 51]. *S. typhimurium* A1-R can possibly overcome de-differentiation of a tumor leading to resistance to targeted chemotherapy, where the targeted protein or pathway may no longer be expressed [51], since *S. typhimurium* A1-R does not depend on such targets [46, 48]. *S. typhimurium* A1-R may also be effectively combined with teratogens which could selectively affect cancer cells that are dedifferentiated [47]. Since *S. typhimurium* A1-R can decoy quiescent cancer cells to begin to cycle, *S. typhimurium* A1-R could be effectively combined with agents which selectively target proliferating cancer cells [49], where normal cells are protected by agents which induce wild type p53 [50].

## MATERIALS AND METHODS

### Mice

Athymic *nu/nu* nude mice (AntiCancer Inc., San Diego, CA), 4–6 weeks old, were used in this study. All animal studies were conducted with an AntiCancer Institutional Animal Care and Use Committee (IACUC)-protocol specifically approved for this study and in accordance with the principals and procedures outlined in the National Institute of Health Guide for the Care and Use of Animals under Assurance Number A3873-1. In order to minimize any suffering of the animals, anesthesia and analgesics were used for all surgical experiments. Animals were anesthetized by subcutaneous injection of a 0.02 ml solution of 20 mg/kg ketamine, 15.2 mg/kg xylazine, and 0.48 mg/kg acepromazine maleate. The response of animals during surgery was monitored to ensure adequate depth of anesthesia. The animals were observed on a daily basis and humanely sacrificed by CO_2_ inhalation when they met the following humane endpoint criteria: prostration, significant body weight loss, difficulty breathing, rotational motion and body temperature drop. The use of animals was necessary to evaluate *S. typhimurium* A1-R efficacy *in vivo*. Animals were housed with no more than 5 per cage. Animals were housed in a barrier facility on a high efficiency particulate arrestance (HEPA)-filtered rack under standard conditions of 12-hour light/dark cycles. The animals were fed an autoclaved laboratory rodent diet.

### Cell line

The human colon cancer cell line HT-29, [52, 53] was maintained in DMEM (Irvine Scientific, Irvine, CA) supplemented with heat-inactivated 10% fetal bovine serum (FBS) (Gemini Biologic Products, Calabasas, CA), 2 mM glutamine, 100 units/ml penicillin, 100 μg/ml streptomycin, and 0.25 μg/ml amphotericin B (Life Technologies, Inc., Grand Island, NY). The cells were incubated at 37°C in 5% CO2.

### Establishment of RFP-labeled HT29

The pDsRed-2 vector (Clontech Laboratories Inc., Palo Alto, CA) expressing RFP and the neomycin-resistance gene was used to stably transfect HT-29 cells to express RFP. For RFP gene transfection, 25% confluent HT-29 cells were incubated with a mixture of retroviral supernatants of PT67-RFP packaging cells and DMEM for 24 h. Fresh medium was replenished at this time, and cells were allowed to grow in the absence of retrovirus for 12 h. This procedure was repeated until high levels of RFP expression were achieved. Cells were then harvested with trypsin-EDTA and subcultured into selective medium that contained G418 (200 μg/ml) (Geneticin, Invitrogen Corp., Carlsbad, CA). The level of G418 was increased to 2,000 μg/ml stepwise. Clones expressing high levels of RFP were isolated and were amplified and transferred using conventional culture methods. High RFP-expression clones were isolated in the absence of G418 for 10 passages to select for stable expression of RFP [52, 54, 55].

### Preparation of *S. typhimurium* A1-R

GFP-expressing *S. typhimurium* A1-R bacteria (AntiCancer Inc.) were grown overnight on LB medium (Fisher Sci., Hanover Park, IL, USA) and then diluted 1:10 in LB medium. Bacteria were harvested at late-log phase, washed with PBS, and then diluted in PBS [23].

### Initial establishment of liver metastases

HT-29-RFP cells were harvested by trypsinization and washed twice with serum-free medium. Cells (5×10^5^ in 50 μl serum-free medium with 50% Matrigel) were injected into the superior and inferior pole of the spleen in mice. Three weeks after injection, liver metastases were established.

### Surgical orthotopic implantation of liver metastasis

Established liver metastases were resected and cut into blocks (3 mm^3^). A single tumor fragment was orthotopically implanted into the left lobe of the liver in other nude mice. Four weeks later, liver metastasis was observed in the implanted site.

### Efficacy for adjuvant *S. typhimurium* A1-R treatment on liver metastasis after resection

Four weeks after orthotopic implantation to the liver, the liver metastasis was resected along the tumor margin under bright light. Twelve mice treated with standard bright-light surgery (BLS) were randomized into 2 groups: a control group (n=6) and an *S. typhimurium* A1-R adjuvant group (n=6). *S. typhimurium* A1-R (5 × 10^7^ CFU/body, iv, weekly, 3 weeks) was administered to the mice as adjuvant treatment beginning one week after BLS. Tumor fluorescence was visualized with the OV100 variable magnification small animal fluorescence imaging system (Olympus Corp., Tokyo, Japan) before and right after BLS. Recurrence was monitored by weekly non-invasive tumor fluorescence evaluation with the LT-9900 Illumatool (Lightools Research, Encinitas, CA, USA) until the end of the experiment.

### Statistical analysis

SPSS statistics (version 21.0) was used for all statistical analyses (IBM, New York City, NY, USA). Survival curves were constructed using the Kaplan–Meier method and compared using the log-rank test. A probability value of *P* < 0.05 was considered statistically significant.
